# Supplementary material for: Recurrent deletions in clonal hematopoiesis are driven by microhomology-mediated end joining
Source: Nat Commun. 2021 Apr 28;12:2455. doi: 10.1038/s41467-021-22803-y (PMC8080710; doi:10.1038/s41467-021-22803-y)
Supplement: Supplementary file 1 — Supplementary Information [file 41467_2021_22803_MOESM1_ESM.pdf]

Supplementary Information

Recurrent deletions in clonal hematopoiesis are driven by Microhomology-mediated end joining

Tzah Feldman[1], Akhiad Bercovich [2*], Yoni Moskovitz [1*], Noa Chapal-Ilani [1], Amanda Mitchell [3], Jessie JF Medeiros [3 4], Tamir Biezuner [1], Nathali Kaushansky [1], Mark D Minden [3 5 6 7], Vikas Gupta [3 6 7], Michael Milyavsky [8 9], Zvi Livneh [10], Amos Tanay [2], Liran I Shlush [❖1 3 11]

[1] Department of Immunology, Weizmann Institute of Science, Rehovot, Israel.
[2] Department of Computer Science and Applied Mathematics, Weizmann Institute of Science, Rehovot, Israel.
[3] Princess Margaret Cancer Centre, University Health Network (UHN) Toronto, Ontario M5G 2M9, Canada.
[4] Department of Molecular Genetics, University of Toronto, Toronto, ON, Canada.
[5] Department of Medical Biophysics, University of Toronto, Toronto, Ontario, Canada.
[6] Department of Medicine, University of Toronto, Toronto, Ontario, Canada.
[7] Division of Medical Oncology and Hematology, University Health Network, Toronto, Ontario, Canada.
[8] Department of Pathology, Tel-Aviv University, Tel-Aviv, 69978, Israel.
[9] Sackler Faculty of Medicine, Tel-Aviv University, Tel-Aviv, 69978, Israel.
[10] Department of Biomolecular Sciences, Weizmann Institute of Science, Rehovot, Israel.
[11] Division of Hematology, Rambam Healthcare Campus, Haifa, Israel.
* denotes equal contribution
❖ corresponding author

Content:
Supplementary Figures 1-12
Supplementary Tables 1-5

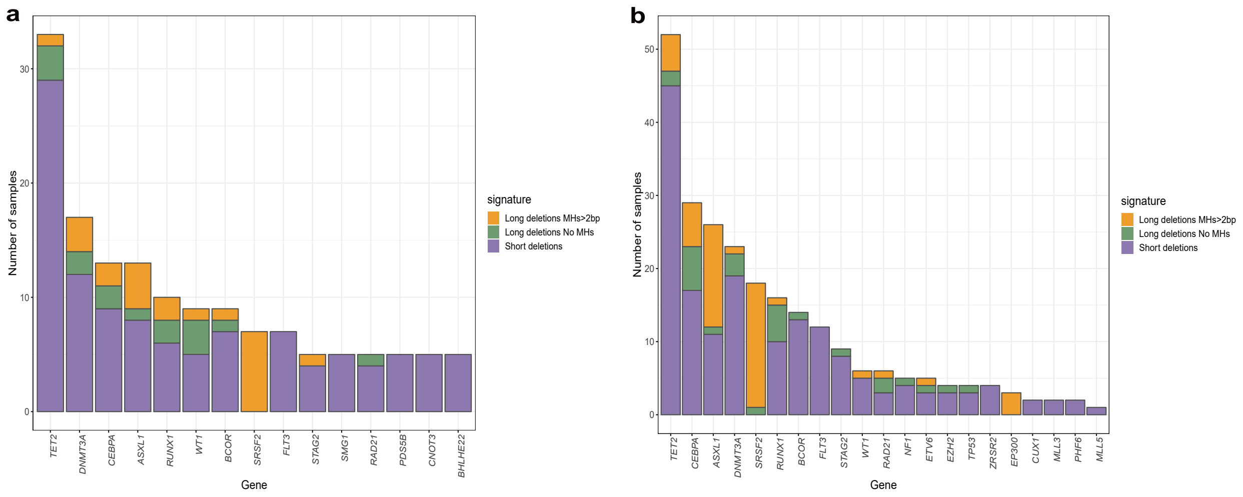

Supplementary Fig. 1 Recurrent mutated genes in AML are enriched with MH-based deletion signatures. **a b,** Number of samples carrying somatic deletions in the most commonly deleted genes from whole-exome sequencing data of 562 AMLs (a) and targeted sequencing data of 1540 adult-AML (b). The proportion of samples containing the different deletion signatures out of the total samples with deletions per gene are showed. Deletion signatures are: ≥5-bp deletions with flanking microhomologies (MHs) of at least 2bp (orange), ≥5-bp deletion with flanking MHs of zero or 1 bp (green) and short deletion (<5-bp) (purple). Source data are provided as a Source Data file.

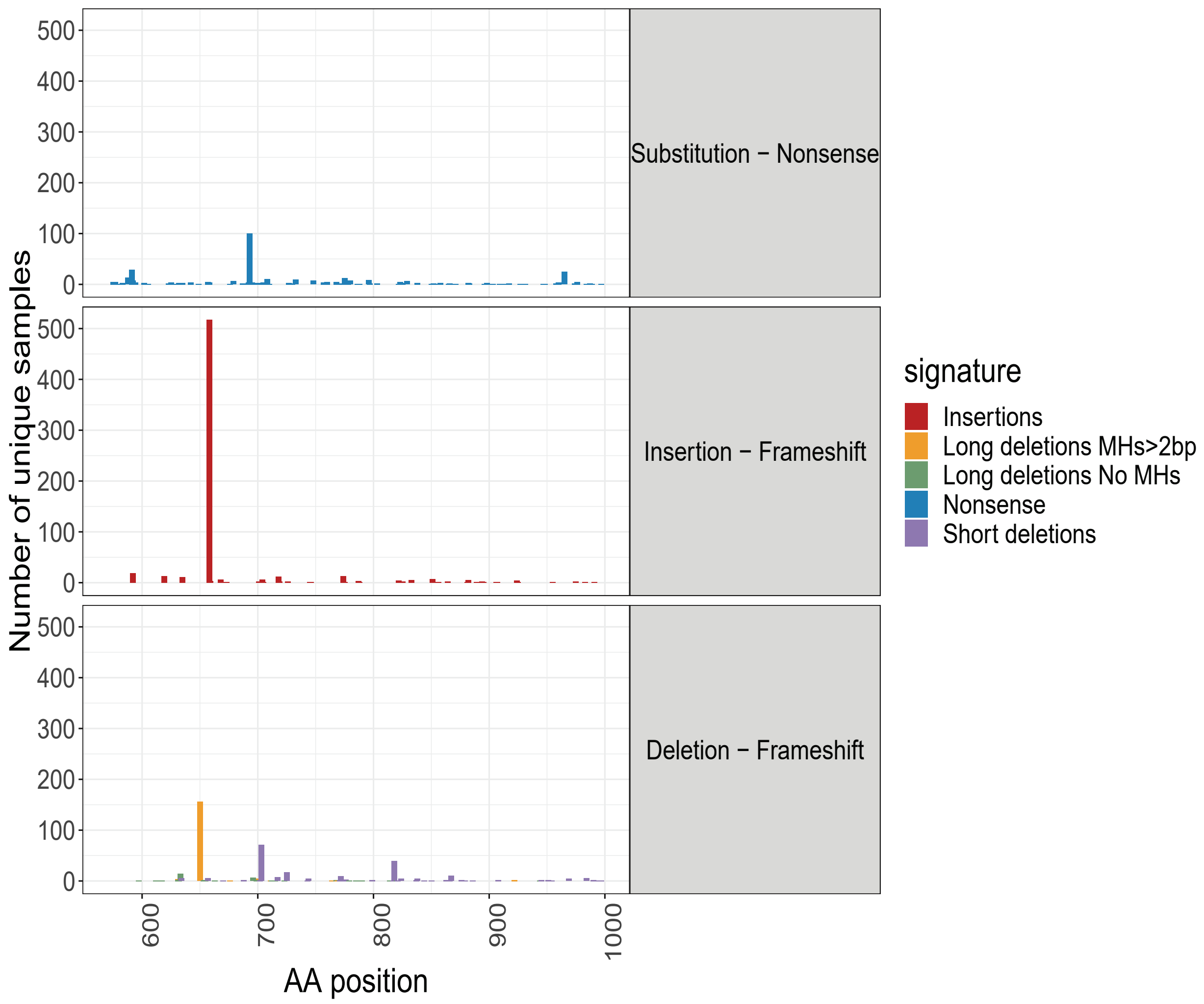

Supplementary Fig. 2 High recurrence rates of *ASXL1* MH-based deletion in myeloid malignancies are driven by specific mutational mechanisms. Number of samples carrying somatic truncating mutations in *ASXL1* gene and the position of the last amino acid of ASXL1 protein (AA position) as identified across *n*=1686 mutated samples from COSMIC dataset. Signatures are Nonsense substitutions (blue), frameshift insertions (red), frameshift ≥5-bp deletions with flanking microhomologies (MHs) of at least 2bp (orange), frameshift ≥5-bp deletions with flanking MHs of zero or 1 bp (green) and frameshift short deletions (<5-bp) (purple). Source data are provided as a Source Data file.

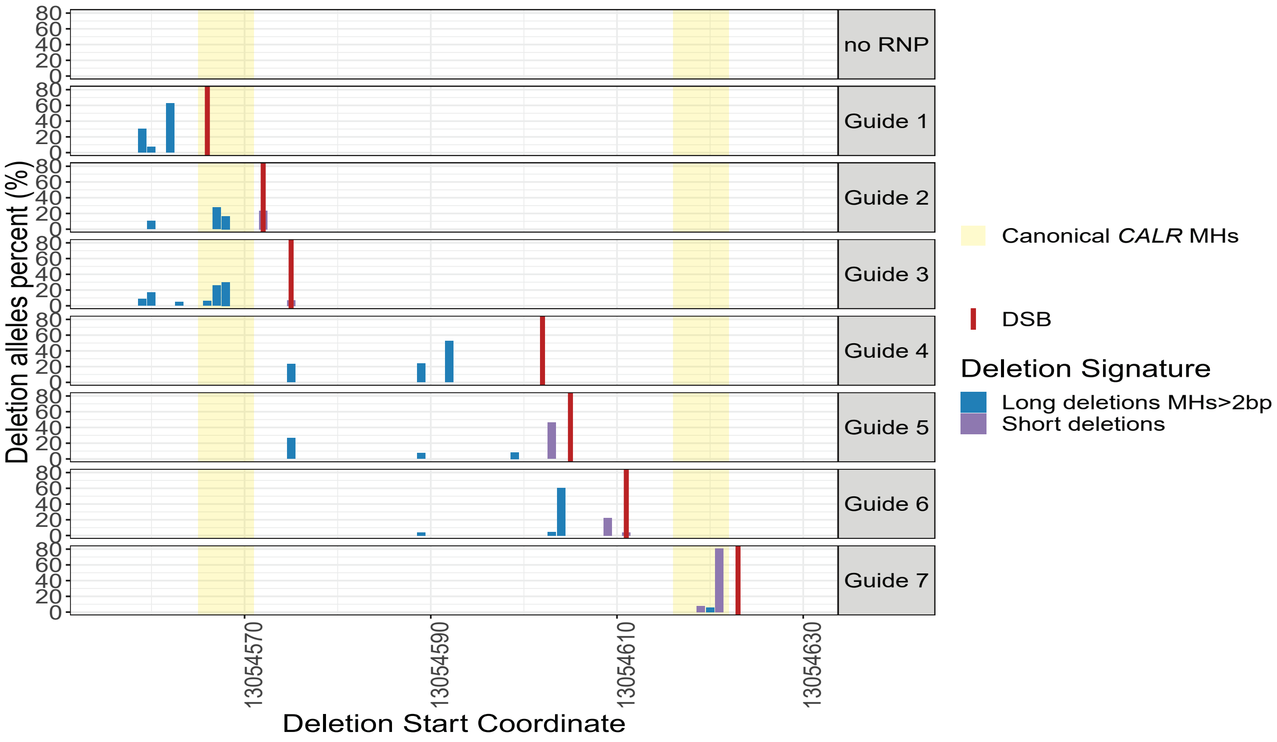

Supplementary Fig. 3 Deletion distribution in *the CALR* gene following sequential CRISPR/Cas9 DSBs in K562 cells. Start genomic positions and percentage of *CALR* deletion alleles among the total deletion alleles obtained for each sgRNA guide as assessed by deep targeted sequencing (read depth 5000X) in the K562 cell line. Insertions are not shown. Deletion signatures are: ≥5-bp deletion with flanking microhomologies (MHs) of at least 2bp (blue), ≥5-bp deletion with flanking MHs of zero or 1 bp (green) and short deletion (<5-bp) (purple). Canonical MHs (yellow backgrounds) and sequential DSBs (vertical red lines) are marked along the *CALR* sequence. 'no RNP' controls represent samples treated with Cas9 in the absence of sgRNAs. Source data are provided as a Source Data file.

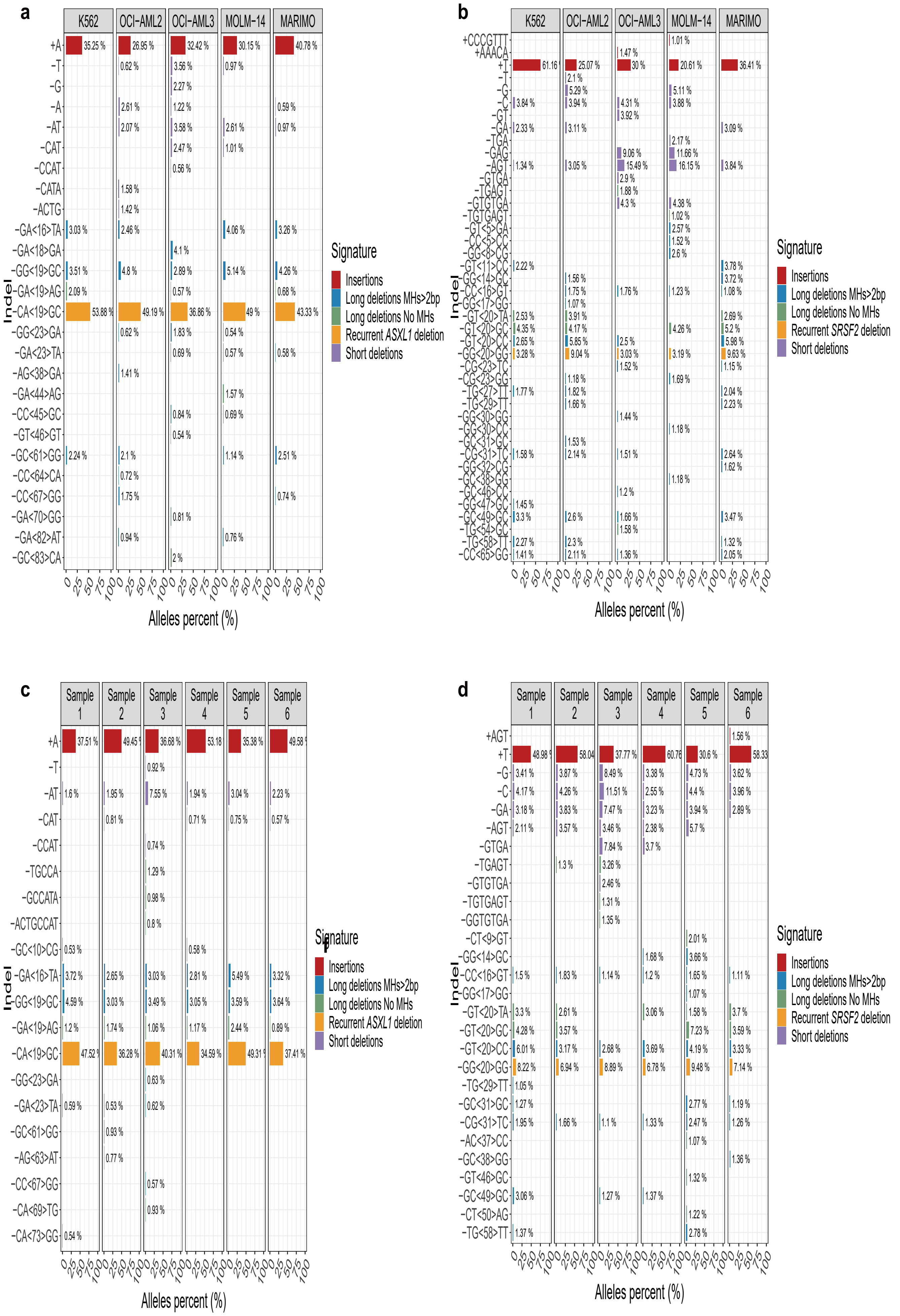
a
K562
OCI-AML2
OCI-AML3
MOLM-14
MARIMO
Indel
Alleles percent (%)
Signature
Insertions
Long deletions MHs>2bp
Long deletions No MHs
Recurrent ASXL1 deletion
Short deletions
b
K562
OCI-AML2
OCI-AML3
MOLM-14
MARIMO
Indel
Alleles percent (%)
Signature
Insertions
Long deletions MHs>2bp
Long deletions No MHs
Recurrent SRSF2 deletion
Short deletions
c
Sample 1
Sample 2
Sample 3
Sample 4
Sample 5
Sample 6
Indel
Alleles percent (%)
Signature
Insertions
Long deletions MHs>2bp
Long deletions No MHs
Recurrent ASXL1 deletion
Short deletions
d
Sample 1
Sample 2
Sample 3
Sample 4
Sample 5
Sample 6
Indel
Alleles percent (%)
Signature
Insertions
Long deletions MHs>2bp
Long deletions No MHs
Recurrent SRSF2 deletion
Short deletions

Supplementary Fig. 4 CRISPR/Cas9 mediated DSBs recapitulate recurrent MH-based deletions in myeloid malignancies. Indel sequences and percentage of *ASXL1* (a, c) and *SRSF2* (b, d) modified alleles among the total indel alleles as assessed by deep targeted sequencing (read depth 5000X) in K562, OCI-AML2, OCI-AML3, MOLM-14 and MARIMO cells (a, b) or primary human CD34+ HSPCs isolated from six individuals (c, d) following the induction of DSBs in *ASXL1* (a, c) and *SRSF2* (b, d) loci. Allele percent of 0.5% (a, c) or 1% (b, d) and above are shown. Deletion signatures are: ≥5-bp deletion with flanking microhomologies (MHs) of at least 2bp (blue), ≥5-bp deletion with flanking MHs of zero or 1 bp (green), short deletion (<5-bp) (purple) and the recurrent deletions in *ASXL1* (a) *SRSF2* (b) genes (orange). Source data are provided as a Source Data file.

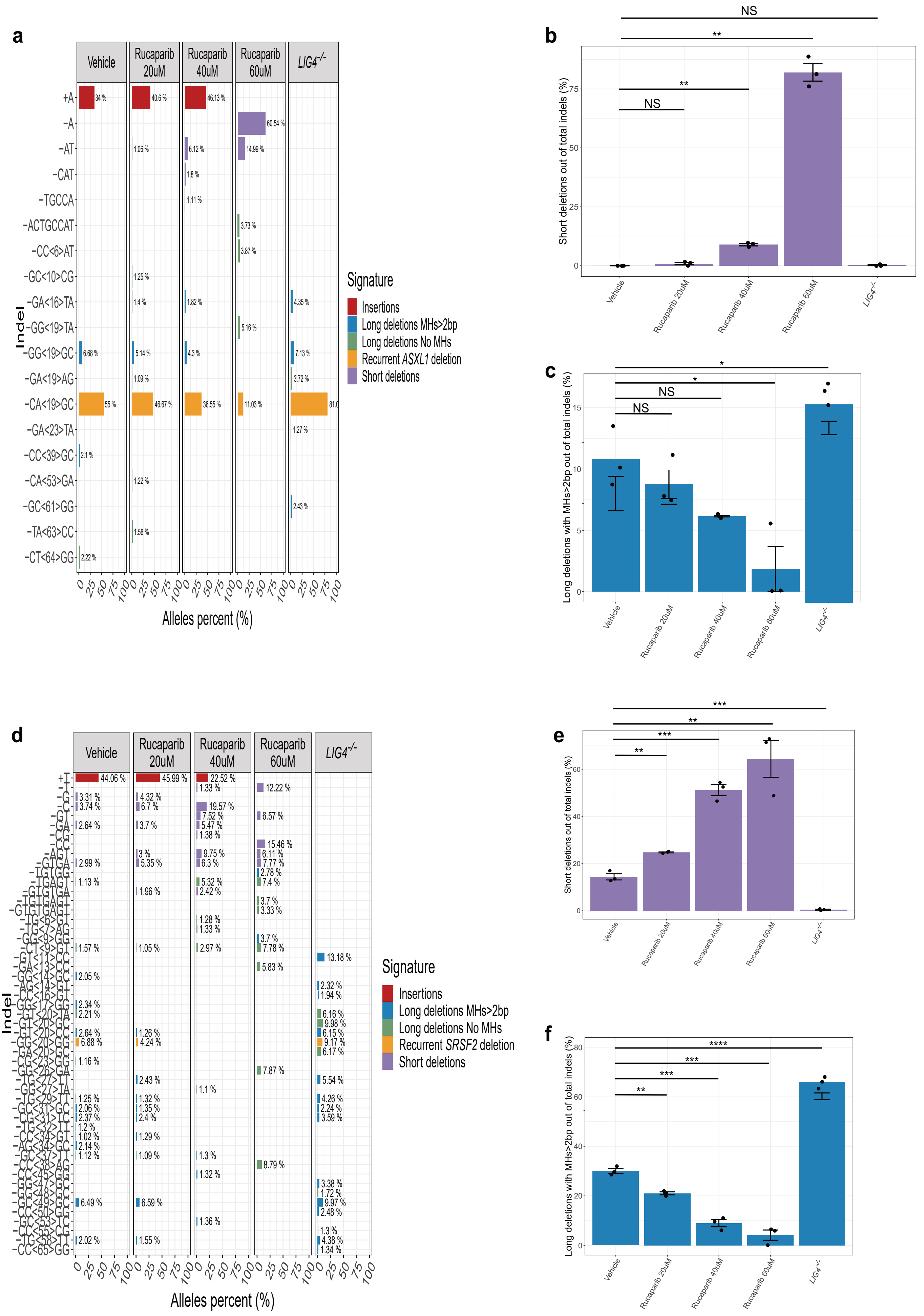

a
Vehicle
Rucaparib 20uM
Rucaparib 40uM
Rucaparib 60uM
LIG4-/-
+A
-A
-AT
-CAT
-TGCCA
-ACTGCCAT
-CC<6>AT
-GC<10>CG
-GA<16>TA
-GG<19>TA
-GG<19>GC
-GA<19>AG
-CA<19>GC
-GA<23>TA
-CC<39>GC
-CA<53>GA
-GC<61>GG
-TA<63>CC
-CT<64>GG
Indel
Alleles percent (%)
Signature
Insertions
Long deletions MHs>2bp
Long deletions No MHs
Recurrent ASXL1 deletion
Short deletions
b
Short deletions out of total indels (%)
NS
**
Vehicle
Rucaparib 20uM
Rucaparib 40uM
Rucaparib 60uM
LIG4-/-
c
Long deletions with MHs>2bp out of total indels (%)
NS
*
d
Indel
Alleles percent (%)
Signature
Insertions
Long deletions MHs>2bp
Long deletions No MHs
Recurrent SRSF2 deletion
Short deletions
e
Short deletions out of total indels (%)
**
***
f
Long deletions with MHs>2bp out of total indels (%)
**
***
****

Supplementary Fig. 5 Recurrent MH-based deletions in myeloid malignancies are the result of *PARP1* mediated MMEJ repair. **a**, **d** Indel sequences and percentage of *ASXL1* (a) and *SRSF2* (d) modified alleles among the total indel alleles as assessed by deep targeted sequencing (read depth 5000X) in K562 cells following the induction of DSBs in *ASXL1* (a) or *SRSF2* (d) loci. *LIG4* -/- K562 cells (right panels) are presented together with Wild type (WT) K562 cells that were electroporated in the presence of the DMSO vehicle, 20, 40 or 60 uM rucaparib camsylate as indicated. Allele percent of 1% (a, d) and above are shown. **b c e f** Percentage of the short deletions (b, e) and long deletions flanked by MHs of at least 2bp (c, f) among the total indel alleles following the induction of DSBs in *ASXL1* (b, c) or *SRSF2* (e, f) loci. WT K562 cells that were electroporated in the presence of the DMSO vehicle, 20, 40 or 60 uM rucaparib camsylate, together with *LIG4* -/- K562 cells are shown. Data are presented as mean values +/- SEM. *n*= 3 biologically independent samples. Unpaired two tailed T-test was used to determine statistical significance. (NS, nonsignificant, $^*P < 0.05$, $^{**}P < 0.01$, $^{***}P < 0.001$, and $^{****}P < 0.0001$). **b**: vehicle vs. rucaparib 20uM p = 0.19, vehicle vs. rucaparib 40uM p = 0.0034, vehicle vs. rucaparib 60uM p = 0.002, vehicle vs. *LIG4* -/- p = 0.42. **c**: vehicle vs. rucaparib 20uM p = 0.32, vehicle vs. rucaparib 40uM p = 0.078, vehicle vs. rucaparib 60uM p = 0.017, vehicle vs. *LIG4* -/- p = 0.023. **e**: vehicle vs. rucaparib 20uM p = 0.0015, vehicle vs. rucaparib 40uM p = 0.00016, vehicle vs. rucaparib 60uM p = 0.0032, vehicle vs. *LIG4* -/- p = 0.0004. **f**: vehicle vs. rucaparib 20uM p = 0.0013, vehicle vs. rucaparib 40uM p = 0.0002, vehicle vs. rucaparib 60uM p = 0.0003, vehicle vs. *LIG4* -/- p = 2.86e-05. Deletion signatures are: ≥5-bp deletion with flanking microhomologies (MHs) of at least 2bp (blue), ≥5-bp deletion with flanking MHs of zero or 1 bp (green), short deletion (<5-bp) (purple) and the recurrent deletions in *ASXL1* (a) *SRSF2* (d) genes (orange). Source data are provided as a Source Data file.

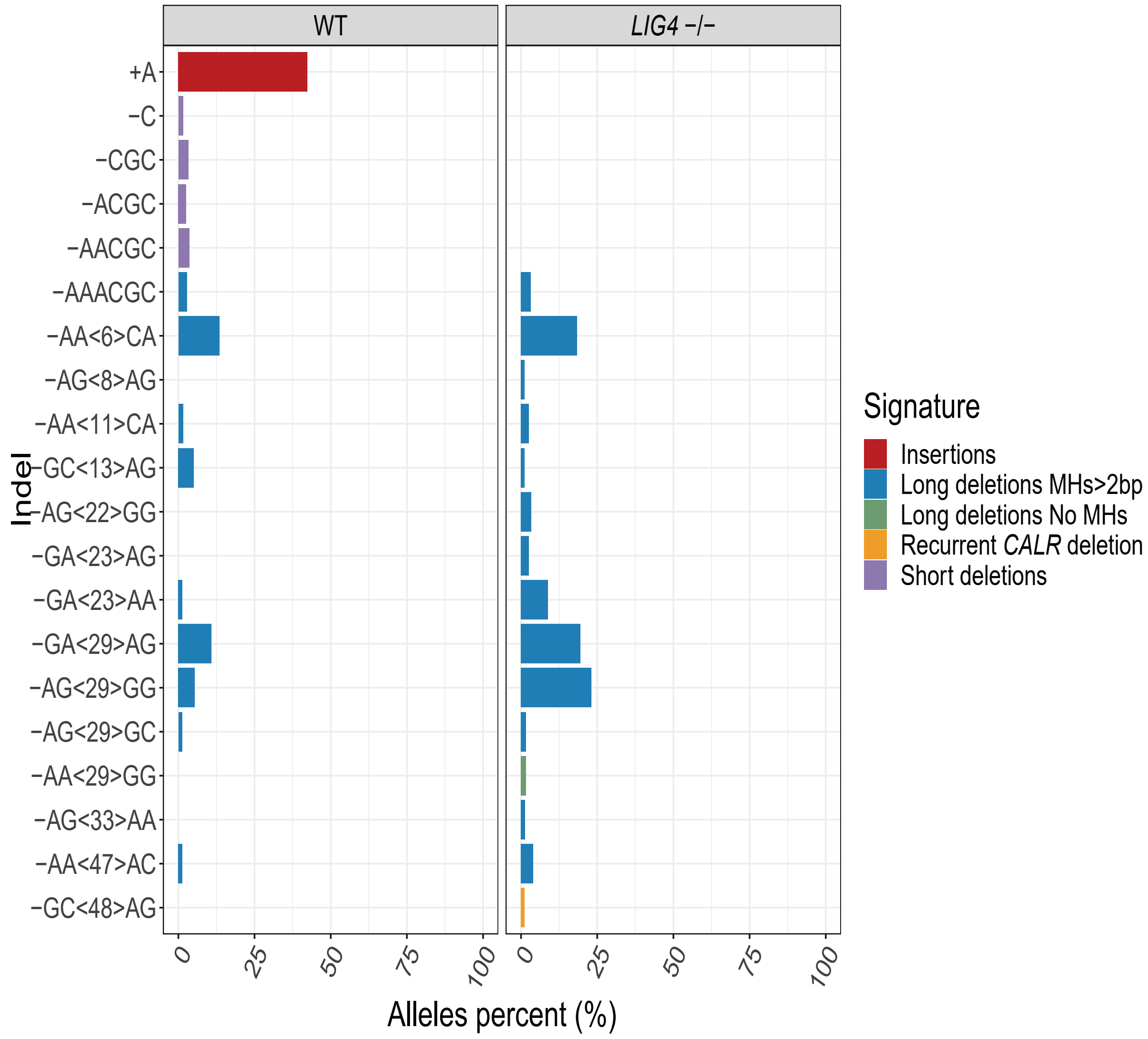

Supplementary Fig. 6 Recurrent MH-based deletion in *CALR* gene is increased in *LIG4* knockout cells. Indel sequences and percentage of *CALR* modified alleles among the total indel alleles as assessed by deep targeted sequencing (read depth 5000X). Wild type (WT) and *LIG4* -/- K562 cells are shown. Indel signatures are: insertion (red), ≥5-bp deletion with flanking microhomologies (MHs) of at least 2bp (blue), ≥5-bp deletion with flanking MHs of zero or 1 bp (green), short deletion (<5-bp) (purple) and the recurrent deletion in *CALR* gene (orange). Source data are provided as a Source Data file.

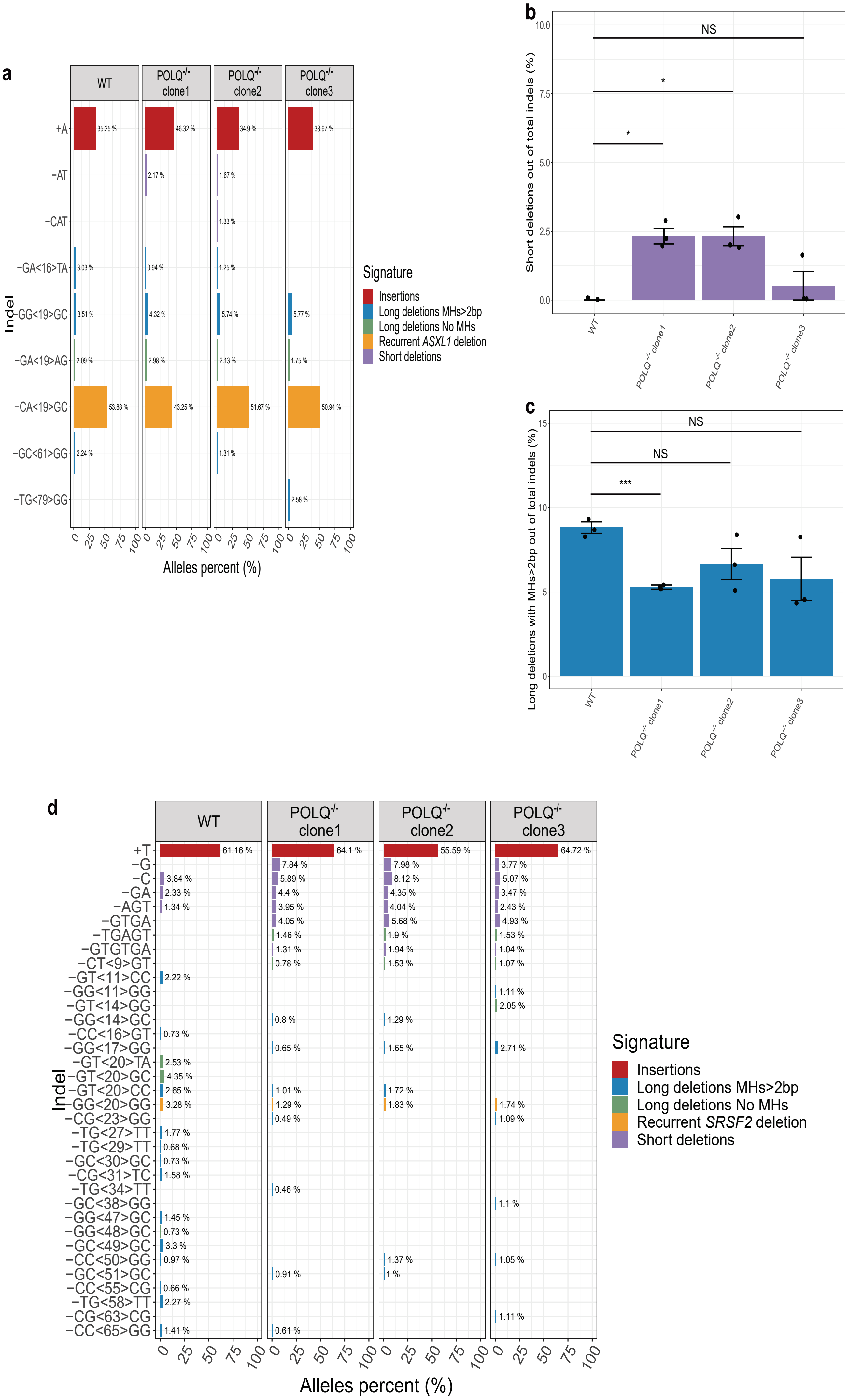

a
WT
POLQ-/- clone1
POLQ-/- clone2
POLQ-/- clone3
+A
−AT
−CAT
−GA<16>TA
−GG<19>GC
−GA<19>AG
−CA<19>GC
−GC<61>GG
−TG<79>GG
Indel
35.25 %
46.32 %
34.9 %
38.97 %
2.17 %
1.67 %
1.33 %
3.03 %
0.94 %
1.25 %
3.51 %
4.32 %
5.74 %
5.77 %
2.09 %
2.98 %
2.13 %
1.75 %
53.88 %
43.25 %
51.67 %
50.94 %
2.24 %
1.31 %
2.58 %
0 25 50 75 100
Alleles percent (%)
Signature
Insertions
Long deletions MHs>2bp
Long deletions No MHs
Recurrent ASXL1 deletion
Short deletions
b
Short deletions out of total indels (%)
10.0
7.5
5.0
2.5
0.0
NS
*
*
WT
POLQ-/- clone1
POLQ-/- clone2
POLQ-/- clone3
c
Long deletions with MHs>2bp out of total indels (%)
15
10
5
0
NS
NS
***
WT
POLQ-/- clone1
POLQ-/- clone2
POLQ-/- clone3
d
WT
POLQ-/- clone1
POLQ-/- clone2
POLQ-/- clone3
+T
−G
−C
−GA
−AGT
−GTGA
−TGAGT
−GTGTGA
−CT<9>GT
−GT<11>CC
−GG<11>GG
−GT<14>GG
−GG<14>GC
−CC<16>GT
−GG<17>GG
−GT<20>TA
−GT<20>GC
−GT<20>CC
−GG<20>GG
−CG<23>GG
−TG<27>TT
−TG<29>TT
−GC<30>GC
−CG<31>TC
−TG<34>TT
−GC<38>GG
−GG<47>GC
−GG<48>GC
−GC<49>GC
−CC<50>GG
−GC<51>GC
−CC<55>CG
−TG<58>TT
−CG<63>CG
−CC<65>GG
Indel
61.16 %
64.1 %
55.59 %
64.72 %
7.84 %
7.98 %
3.77 %
3.84 %
5.89 %
8.12 %
5.07 %
2.33 %
4.4 %
4.35 %
3.47 %
1.34 %
3.95 %
4.04 %
2.43 %
4.05 %
5.68 %
4.93 %
1.46 %
1.9 %
1.53 %
1.31 %
1.94 %
1.04 %
0.78 %
1.53 %
1.07 %
2.22 %
1.11 %
2.05 %
0.8 %
1.29 %
0.73 %
0.65 %
1.65 %
2.71 %
2.53 %
4.35 %
2.65 %
1.01 %
1.72 %
3.28 %
1.29 %
1.83 %
1.74 %
0.49 %
1.09 %
1.77 %
0.68 %
0.73 %
1.58 %
0.46 %
1.1 %
1.45 %
0.73 %
3.3 %
0.97 %
1.37 %
1.05 %
0.91 %
1 %
0.66 %
2.27 %
1.11 %
1.41 %
0.61 %
0 25 50 75 100
Alleles percent (%)
Signature
Insertions
Long deletions MHs>2bp
Long deletions No MHs
Recurrent SRSF2 deletion
Short deletions

Supplementary Fig. 7 preL-MMEJ deletions are obtained in *POLQ* knockout cells. **a**, **d** Indel sequences and percentage of *ASXL1* (a) and *SRSF2* (d) modified alleles among the total indel alleles as assessed by deep targeted sequencing (read depth 5000X) in K562 cells following the induction of DSBs in *ASXL1* (a) or *SRSF2* (d) loci. Wild type (WT) K562 together with three distinct clones of *POLQ* -/- K562 cells are shown. **b, c** Percentage of <5-bp short deletions in *ASXL1* (b) and ≥5-bp deletions with flanking microhomologies (MHs) of at least 2bp in *ASXL1* (c) among the total indel alleles following the induction of DSBs in *ASXL1* locus. WT K562 together with three distinct clones of *POLQ* -/- K562 cells are shown. Data are presented as mean values +/- SEM. *n*= 3 biologically independent samples. Unpaired two tailed T-test was used to determine statistical significance. (NS, nonsignificant, *$P < 0.05$, ***$P < 0.001$). **b**: WT vs. *POLQ -/-* clone 1 $p = 0.014$, WT vs. *POLQ -/-* clone 2 $p = 0.021$, WT vs. *POLQ -/-* clone 3 $p = 0.42$. **c**: WT vs. *POLQ -/-* clone 1 $p = 0.0005$, WT vs. *POLQ -/-* clone 2 $p = 0.092$, WT vs. *POLQ -/-* clone 3 $p = 0.083$.Deletion signatures are: ≥5-bp deletion with flanking microhomologies (MHs) of at least 2bp (blue), ≥5-bp deletion with flanking MHs of zero or 1 bp (green), short deletion (<5-bp) (purple) and the recurrent deletions in *ASXL1* (a) *SRSF2* (d) genes (orange). Source data are provided as a Source Data file.

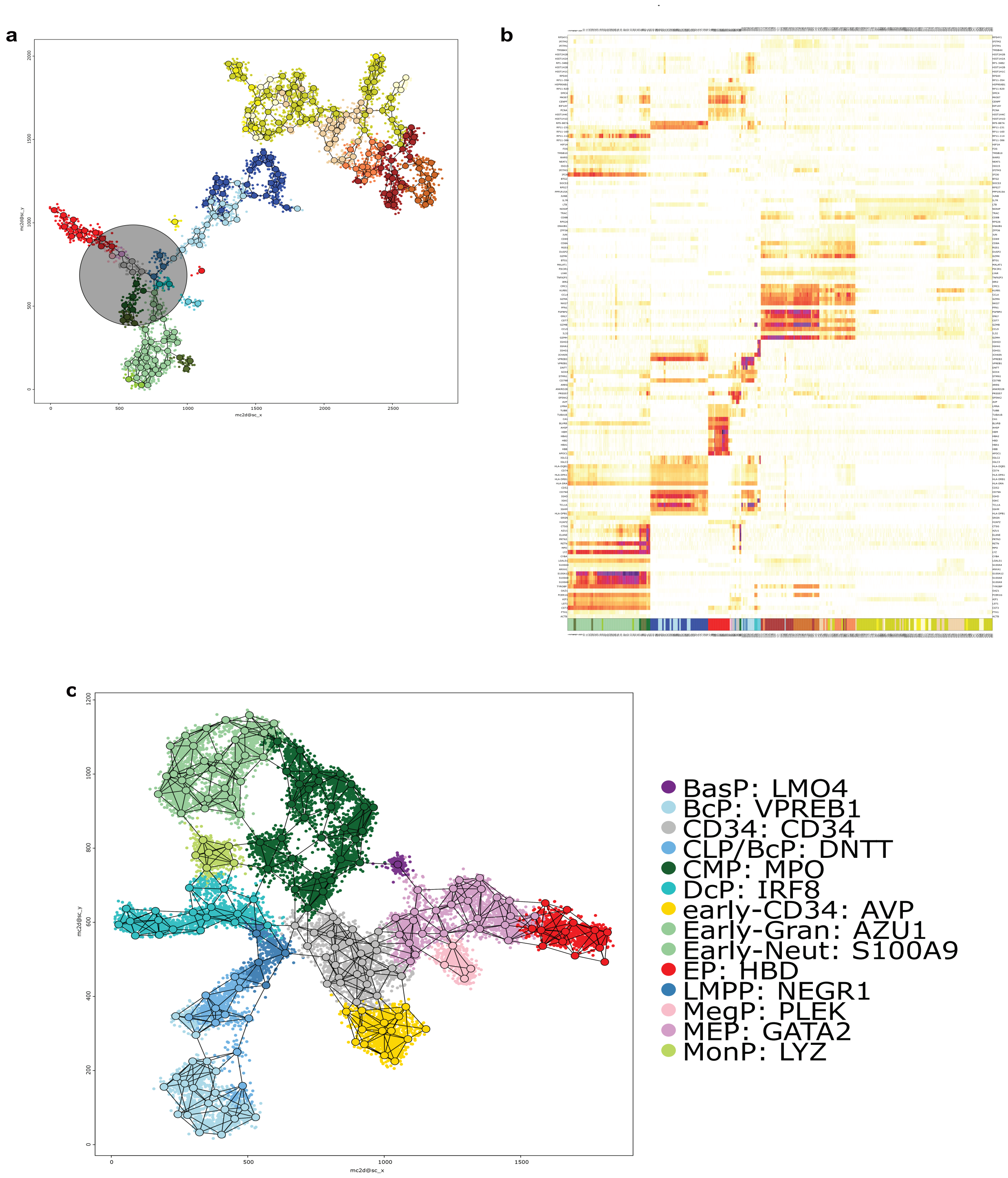

Supplementary Fig. 8 Single cell RNA-seq from all Meta cell model. **a,** 2D projection of annotated metacells after filtering for HSPCs from the entire Human Cell Atlas Consortium's immune census dataset which consists of roughly 310,000 single-cell RNA profiles from adult human BM. **b,** markers defining the different metacells. **c,** 2D projection of annotated metacells after filtering for HSPCs from the full HCA immune census dataset. Markers used for annotation are shown to the right. BasP, basophil progenitor; BcP, B-cell progenitor; CLP, common lymphoid progenitor; CMP, common myeloid progenitor; DcP, dendritic cell progenitor; Early-Gran; early granulocyte; Early-Neut, early neutrophil; EP, erythroid progenitor; LMPP, lymphoid-primed multipotential progenitors; MegP, megakaryocyte progenitor; MEP, megakaryocyte erythrocyte progenitor; MonP, monocyte progenitor; TcP, T-cell progenitor.

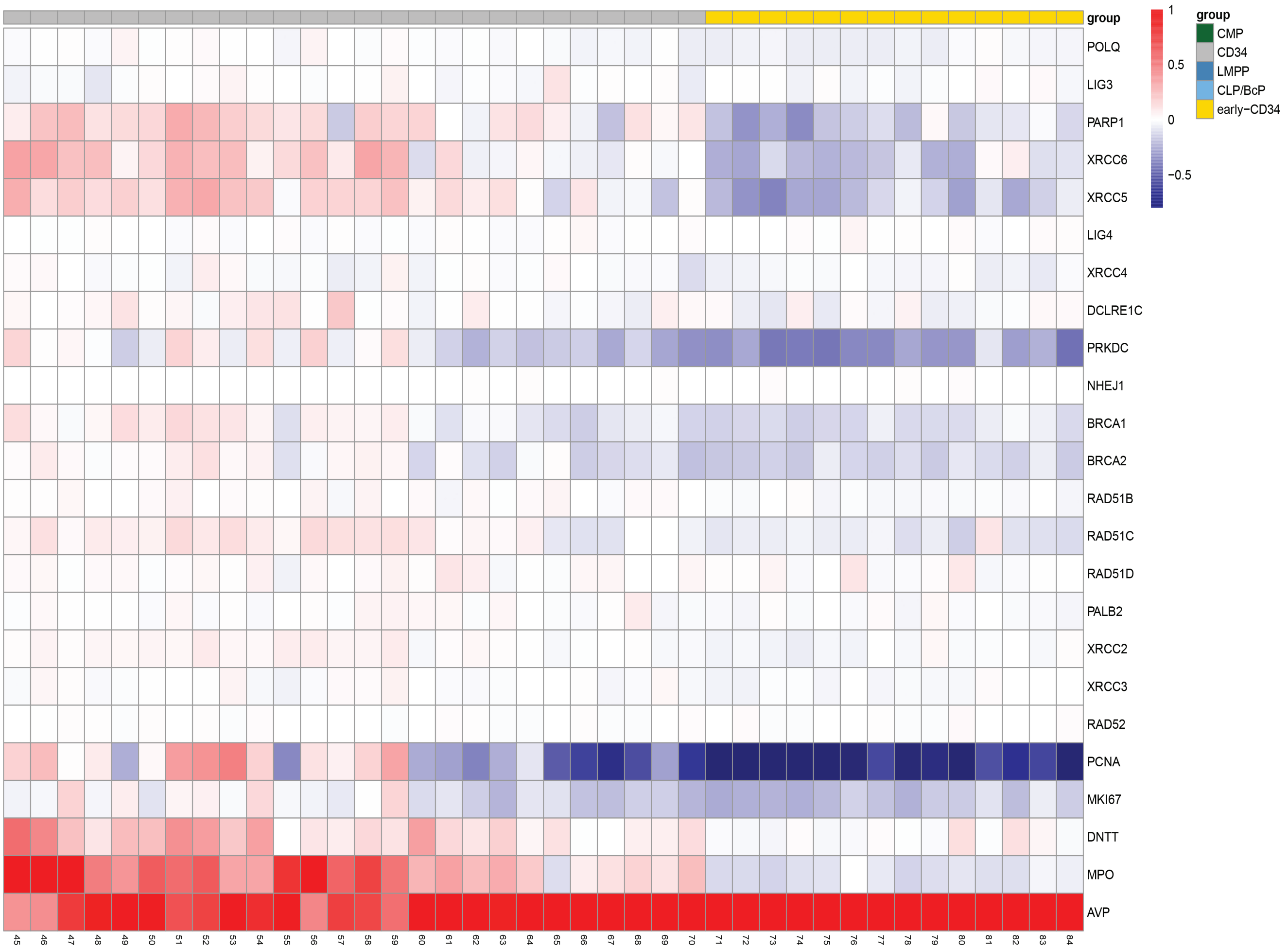

Supplementary Fig. 9 Gene expression levels of DSB repair genes in early HSCs Meta cells. Log fold change (lfp) in UMI content for DSBs repair genes in early HSPCs metacells. Genes markers of proliferation (PCNA and MKI67) and gene markers (MPO, DNTT, AVP) lfp's are also shown.

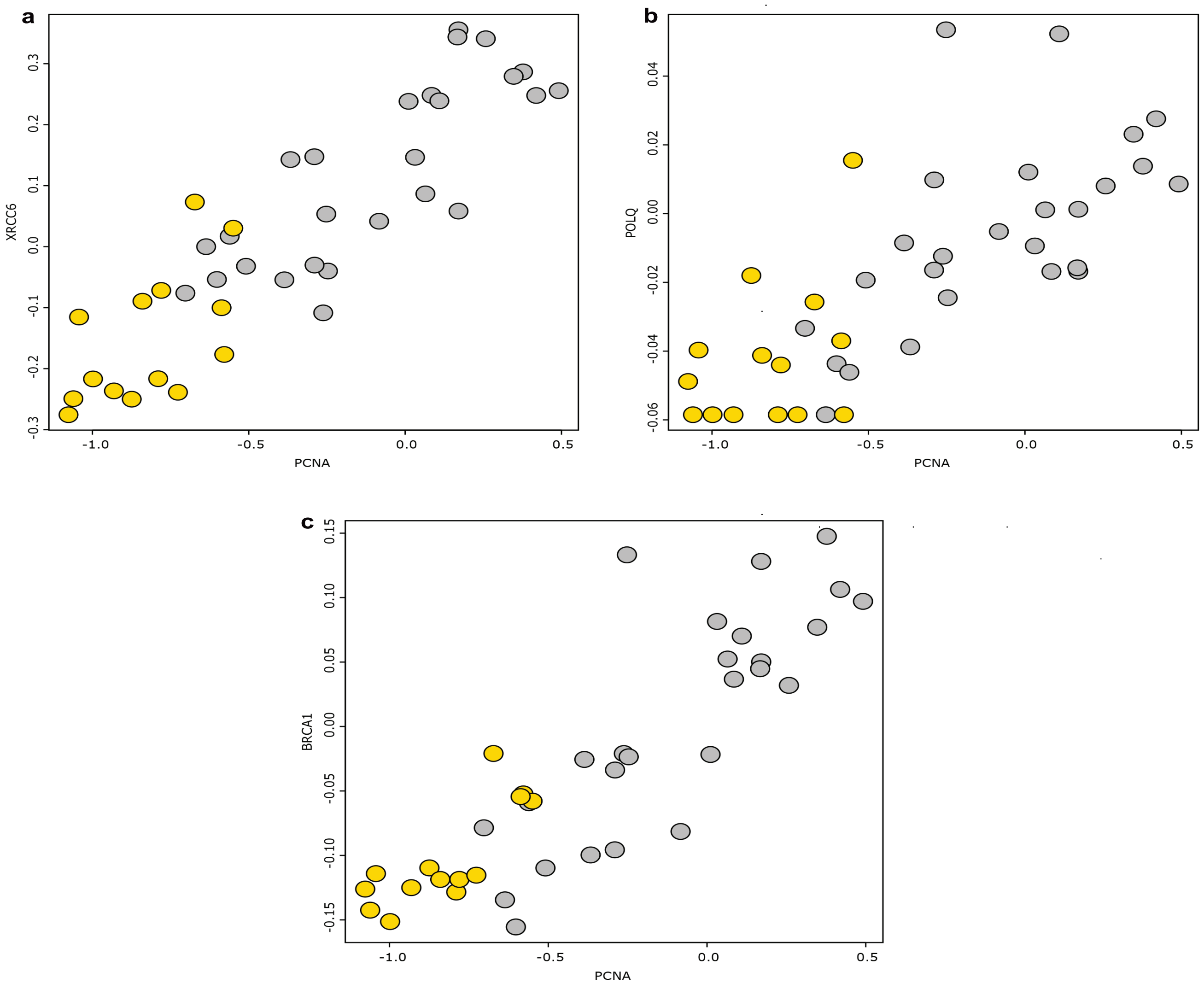

Supplementary Fig. 10 Gene expression levels of key players in c-NHEJ, MMEJ and HR pathways correlate with cell replication in early HSCs. **a b c,** Scatter plots of early HSPCs metacells' lfp values for *XRCC6* (a), *POLQ* (b) and *BRCA1* (c) genes vs. proliferation marker (PCNA).

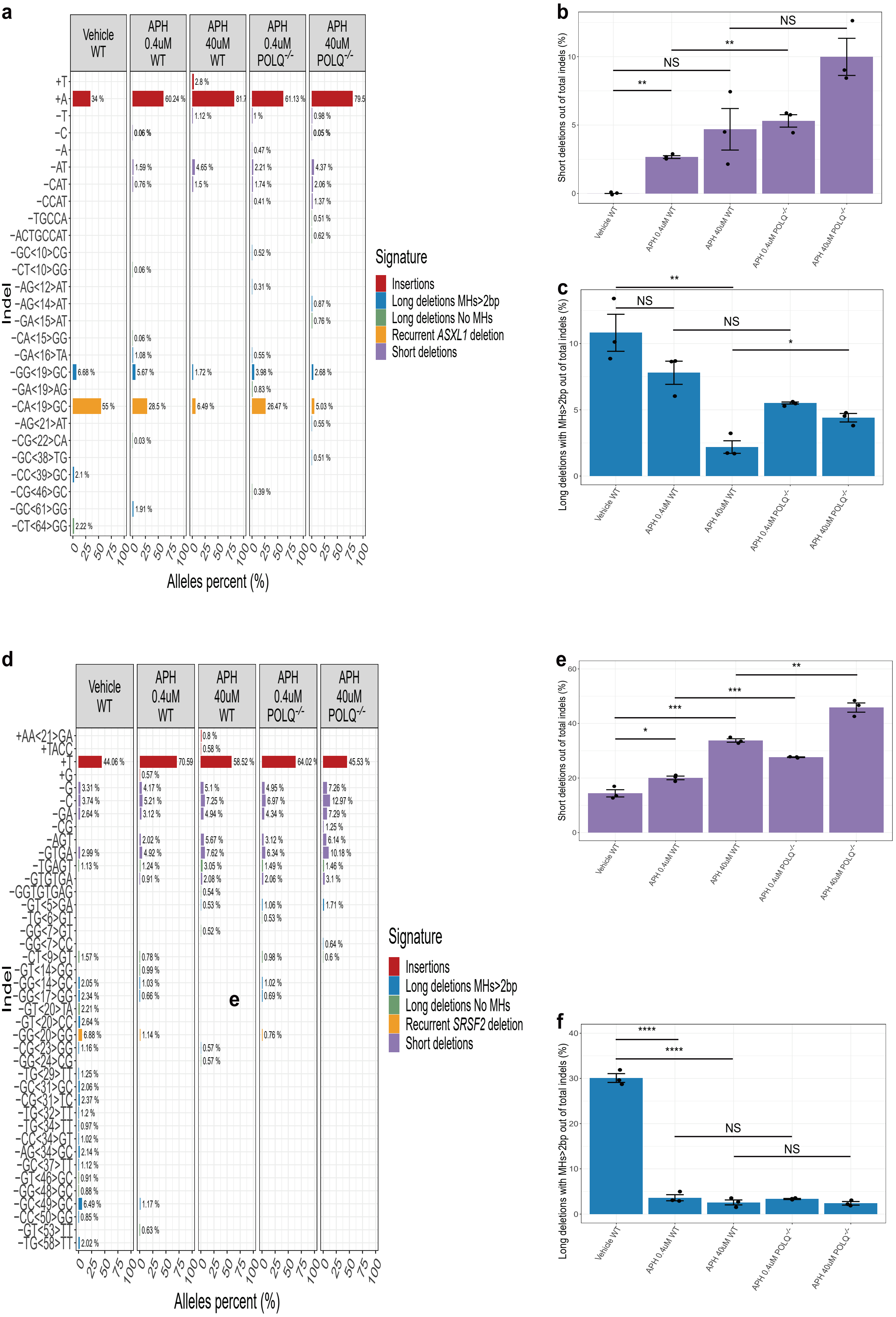
a
Vehicle WT
APH 0.4uM WT
APH 40uM WT
APH 0.4uM POLQ-/-
APH 40uM POLQ-/-
Indel
Alleles percent (%)
Signature
Insertions
Long deletions MHs>2bp
Long deletions No MHs
Recurrent ASXL1 deletion
Short deletions
b
Short deletions out of total indels (%)
c
Long deletions with MHs>2bp out of total indels (%)
d
Vehicle WT
APH 0.4uM WT
APH 40uM WT
APH 0.4uM POLQ-/-
APH 40uM POLQ-/-
Indel
Alleles percent (%)
Signature
Insertions
Long deletions MHs>2bp
Long deletions No MHs
Recurrent SRSF2 deletion
Short deletions
e
Short deletions out of total indels (%)
f
Long deletions with MHs>2bp out of total indels (%)

Supplementary Fig. 11 Aphidicolin treatment reduces the formation of preL-MMEJ deletions. **a d,** Indel sequences and percentage of *ASXL1* (a) and *SRSF2* (d) modified alleles among the total indel alleles as assessed by deep targeted sequencing (read depth 5000X) in K562 cells following the induction of DSBs in *ASXL1* (a) or *SRSF2* (d) loci. Wild type (WT) or *POLQ* -/- K562 cells that were electroporated in the presence of the DMSO vehicle (only WT), 0.4 or 40 uM aphidicolin (APH) are shown. In **d,** allele percent of 0.5% and above are shown. **b c e f,** Percentage of the short deletions (b, e) and long deletions flanked by MHs of at least 2bp (c, f) among the total indel alleles following the induction of DSBs in *ASXL1* (b, c) or *SRSF2* (e, f) loci. WT or *POLQ* -/- K562 cells that were electroporated in the presence of the DMSO vehicle (only WT), 0.4 or 40 uM aphidicolin (APH) are shown. Data are presented as mean values +/- SEM. *n*=3 biologically independent samples. Unpaired two tailed T-test was used to determine statistical significance. (NS, nonsignificant, *$P < 0.05$, **$P < 0.01$, ***$P < 0.001$, and ****$P < 0.0001$). **b**: vehicle WT vs. APH 0.4 uM WT $p = 0.0014$, vehicle WT vs. APH 40 uM WT $p = 0.09$, APH 0.4 uM WT vs. APH 0.4 uM *POLQ -/-* $p = 0.0047$, APH 40 uM WT vs. APH 40 uM *POLQ -/-* $p = 0.06$. **c**: vehicle WT vs. APH 0.4 uM WT $p = 0.14$, vehicle WT vs. APH 40 uM WT $p = 0.004$, APH 0.4 uM WT vs. APH 0.4 uM *POLQ -/-* $p = 0.11$, APH 40 uM WT vs. APH 40 uM *POLQ -/-* $p = 0.018$. **e**: vehicle WT vs. APH 0.4 uM WT $p = 0.018$, vehicle WT vs. APH 40 uM WT $p = 0.00018$, APH 0.4 uM WT vs. APH 0.4 uM *POLQ -/-* $p = 0.0003$, APH 40 uM WT vs. APH 40 uM *POLQ -/-* $p = 0.0025$. **f**: vehicle WT vs. APH 0.4 uM WT $p = 2.37e-05$, vehicle WT vs. APH 40 uM WT $p = 1.61e-05$, APH 0.4 uM WT vs. APH 0.4 uM *POLQ -/-* $p = 0.7$, APH 40 uM WT vs. APH 40 uM *POLQ -/-* $p = 0.78$. Deletion signatures are: ≥5-bp deletion with flanking microhomologies (MHs) of at least 2bp (blue), ≥5-bp deletion with flanking MHs of zero or 1 bp (green), short deletion (<5-bp) (purple) and the recurrent deletions in *ASXL1* (a) *SRSF2* (d) genes (orange). Source data are provided as a Source Data file.

a *LIG4* -/-

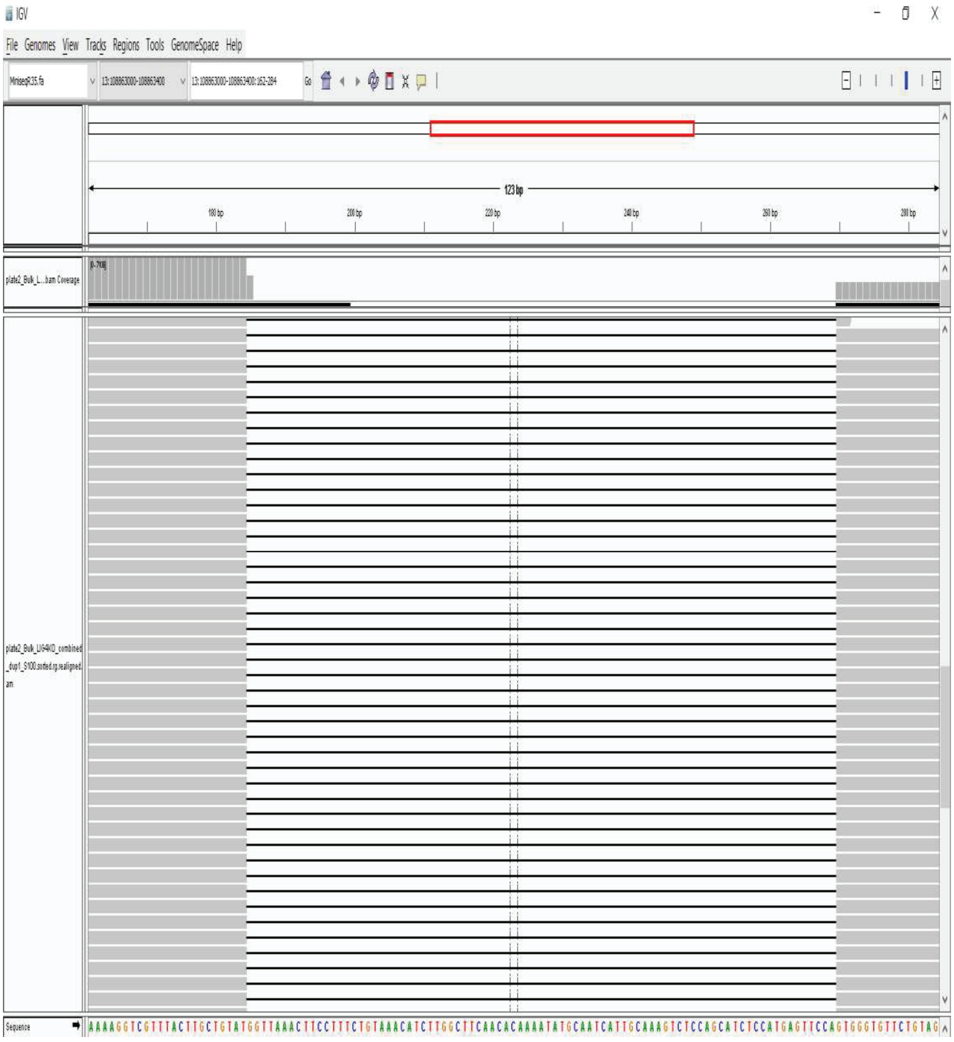

b *POLQ* -/- clone 1 exon 14

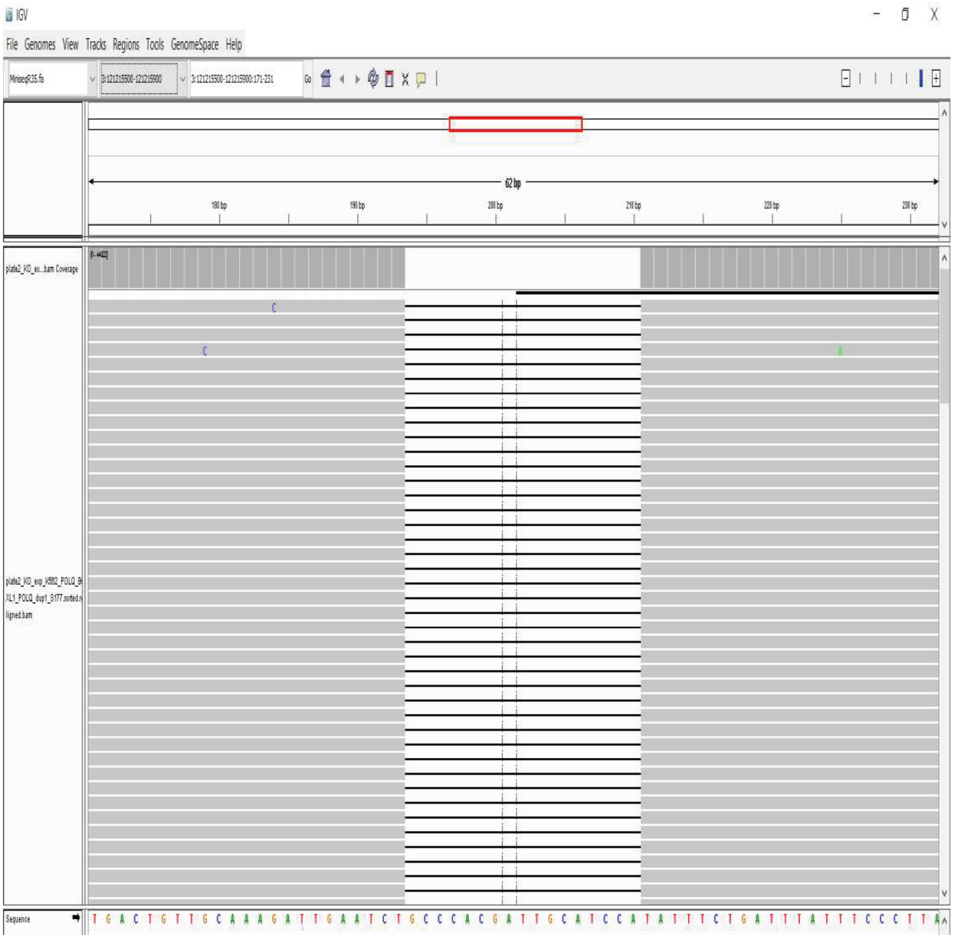

c *POLQ* -/- clone 2 exon 16

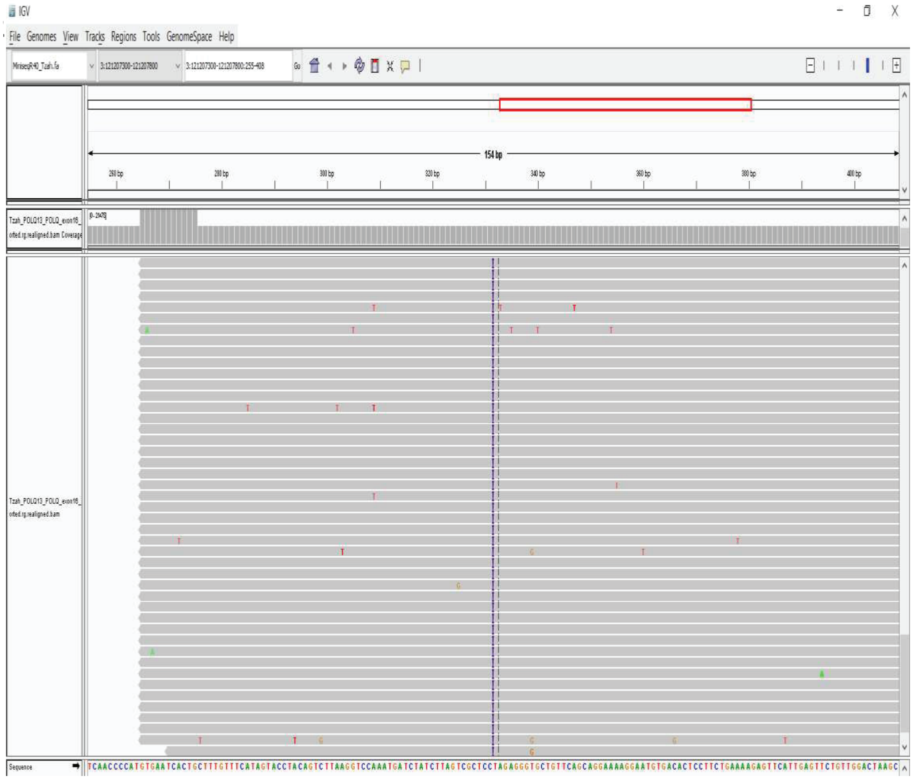

d *POLQ* -/- clone 3 exon 18

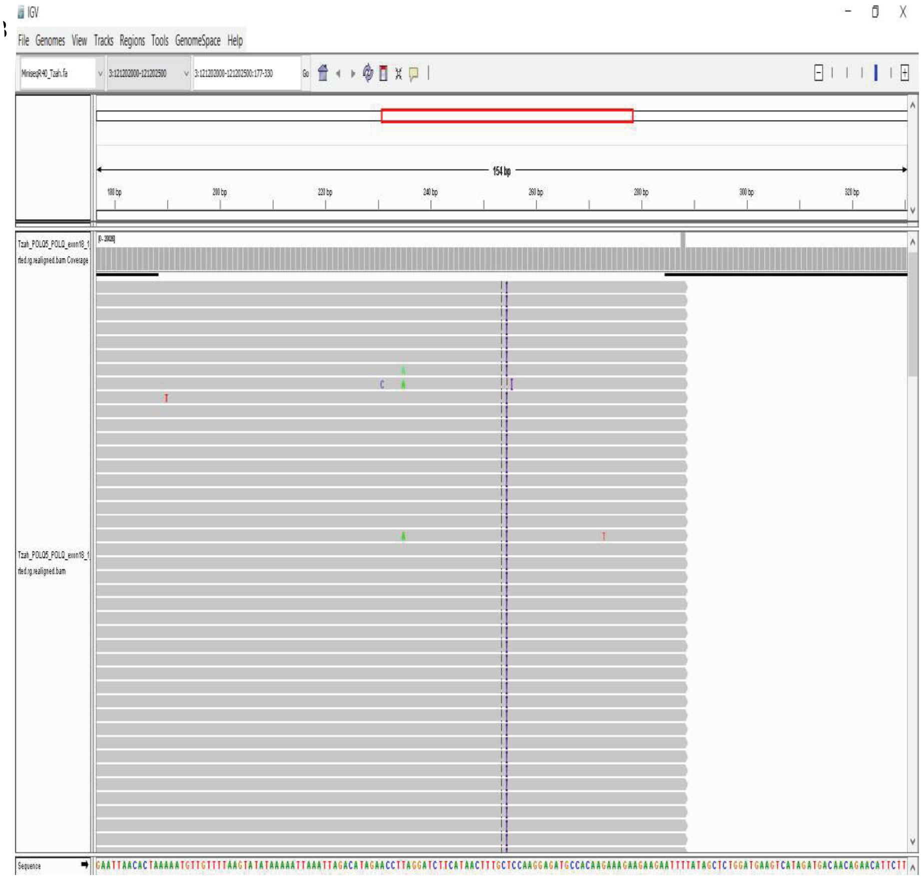

Supplementary Fig. 12 Bi-allelic frame-shift mutations in isolated *LIG4* and *POLQ* knockout cells. **a b c,** Representative IGV images of LIG4 -/- (a) and three *POLQ* -/- (b-d), demonstrating bi-allelic frameshift deletions (a, b) or insertions (c, d) at the gene of interest, leading to premature stop codons.

Sample ID	Chromosome	Position	WT	MT	VAF	Gene	Protein	Effect	Group	Gender	Age at blood sampling	Follow-up length (Years)
EPIC_0062	20	31022403	CACCACTGCCATAGAGAGGCGGC	-	0.1784	ASXL1	p.E635fs*15	Frameshift	pre-AML	Male	64.88	1.78
EPIC_0448	20	31022403	CACCACTGCCATAGAGAGGCGGC	-	0.0483	ASXL1	p.E635fs*15	Frameshift	pre-AML	Male	71.02	8.81
EPIC_0067	20	31022403	CACCACTGCCATAGAGAGGCGGC	-	0.0048	ASXL1	p.E635fs*15	Frameshift	pre-AML	Female	66.51	10.74

Supplementary Table 1. Clinical and genotypic characteristics of three pre-AML samples containing preL-MMEJ deletions in *ASXL1* gene.

Sample no.	Sample Id	Diagnosis	BM Blasts (%)	NPM1	FLT3-ITD	FLT3-TKD	CALR	Jak2 V617f	Bcr Abl1	Cytogenetics
1	282068	AML, NOS (favor M4)	33	UD	UD	UD				47,XY,+8[19]/46,XY[1]
2	162229	AML, NOS	82	UD	UD					48,XX,+13,+13[9]/46,XX[2]
3	161153	AML MRC	44	UD	Positive		UD	UD	UD	46,XX[20]
4	843881	AML MRC	20	UD	UD	UD				47,XX,+8[4]/46,XX[2]
5	167198	PET-MF (ET in Aug 2004, progression to MF in Feb 2017, accelerated phase in April 2017, AML transformation in Jan 2019)	3				Positive	UD	UD	46,XX[20]
	140122	PMF, overt fibrotic	5				Positive	UD	UD	46,XY,del(13)(q12q14)[6]/46,XY[7]
	140681	PET-MF	not done				UD	UD	UD	not done

Supplementary Table 2. Clinical and genotypic characteristics of five AML and two MF samples containing preL-MMEJ deletions. AML, Acute Myeloid Leukemia; MF, Myelofibrosis; PMF, Primary Myelofibrosis; PET, Primary Essential Thrombocytosis; AML-MRC, Acute myeloid leukemia with myelodysplasia-related changes; AML-NOS, AML, not otherwise specified; BM, Bone-marrow; Dx, Diagnosis; UD, Undetectable.

Pateint no.	Sample type	Chromosome	Position	WT	MT	VAF	Gene	Protein	Effect
1	AML cells	17	74732935	GGCGGCTGTGGTGTGAGTCCGGGG	-	0.3200	SRSF2	p.P95_R102del	Inframe
1	T cells	17	74732935	GGCGGCTGTGGTGTGAGTCCGGGG	-	0.0103	SRSF2	p.P95_R102del	Inframe
2	AML cells	17	74732935	GGCGGCTGTGGTGTGAGTCCGGGG	-	0.4900	SRSF2	p.P95_R102del	Inframe
2	T cells	17	74732935	GGCGGCTGTGGTGTGAGTCCGGGG	-	0.0509	SRSF2	p.P95_R102del	Inframe
2	AML cells	20	31022403	CACCACTGCCATAGAGAGGCGGC	-	0.3230	ASXL1	p.E635fs*15	Frameshift
2	T cells	20	31022403	CACCACTGCCATAGAGAGGCGGC	-	0.0267	ASXL1	p.E635fs*15	Frameshift
3	AML cells	20	31022403	CACCACTGCCATAGAGAGGCGGC	-	0.5320	ASXL1	p.E635fs*15	Frameshift
3	T cells	20	31022403	CACCACTGCCATAGAGAGGCGGC	-	0.0145	ASXL1	p.E635fs*15	Frameshift
4	AML cells	20	31022403	CACCACTGCCATAGAGAGGCGGC	-	0.5384	ASXL1	p.E635fs*15	Frameshift
4	T cells	20	31022403	CACCACTGCCATAGAGAGGCGGC	-	0.0208	ASXL1	p.E635fs*15	Frameshift
5	AML cells	20	31022403	CACCACTGCCATAGAGAGGCGGC	-	0.5380	ASXL1	p.E635fs*15	Frameshift
5	T cells	20	31022403	CACCACTGCCATAGAGAGGCGGC	-	0.0490	ASXL1	p.E635fs*15	Frameshift

Supplementary Table 3. Variant allele frequencies of paired AML and T-cells derived from five AML patients containing preL-MMEJ deletions.

Sample no.	Sample Id	Recipient Diagnosis
1	141509	NHL
2	141511	NHL
3	141180	Amyloidosis with Waldenstrom' s macroglobulinemia
4	141182	MM
5	141503	MM
6	141496	NHL

Supplementary Table 4. Clinical characteristics of six patients from which peripheral blood autologous transplant products were obtained. MM, Multiple Myeloma; NHL, Non-Hodgkin Lymphoma.

a

gene	guide	sequence
ASXL1	Guide 1	CAGTGGTGGCCGCCTCTCTA
ASXL1	**Guide 2**	**AGGTCACCACTGCCATAGAG**
ASXL1	Guide 3	TGGCCGCCTCTCTATGGCAG
ASXL1	Guide 4	TCACCACTGCCATAGAGAGG
ASXL1	Guide 5	CGGCCACCACTGCCATCGGA
SRSF2	Guide 1	TGCGGGGTGGCGGTCCCCGG
SRSF2	Guide 2	GACTCACACCACAGCCGCCG
SRSF2	Guide 3	CGGACTCACACCACAGCCGC
SRSF2	Guide 4	GGCGGCTGTGGTGTGAGTCC
SRSF2	**Guide 5**	**GCGGCTGTGGTGTGAGTCCG**
SRSF2	Guide 6	CGGCTGTGGTGTGAGTCCGG
SRSF2	Guide 7	CGGGTGCAAATGGCGCGCTA
CALR	Guide 1	ACAAACAGGACGAGGAGCAG
CALR	Guide 2	GGACGAGGAGCAGAGGCTTA
CALR	Guide 3	CGAGGAGCAGAGGCTTAAGG
CALR	Guide 4	AGAAGACAAGAAACGCAAAG
CALR	Guide 5	AGACAAGAAACGCAAAGAGG
CALR	Guide 6	GAAACGCAAAGAGGAGGAGG
CALR	Guide 7	GGAGGAGGAGGCAGAGGACA

b

gene	sequence
LIG4	GCATCTCCATGAGTTCCAGT
POLQ exon 14	AGAAATATGGATGCAATCGT
POLQ exon 16	CTATCTTAGTCGCTCCTAGA
POLQ exon 18	ATCTTCATAACTTTGCTCCA

c

Gene	Primer Orientation	Universal 5' prefix	Target specific sequence	Final primer
ASXL1	Forward	CTACACGACGCTCTTCCGATCT	accctcgcagacattaaagc	CTACACGACGCTCTTCCGATCTaccctcgcagacattaaagc
ASXL1	Reverse	CAGACGTGTGCTCTTCCGATCT	gtagatctgacgtacactttcca	CAGACGTGTGCTCTTCCGATCTgtagatctgacgtacactttcca
SRSF2	Forward	CTACACGACGCTCTTCCGATCT	ctcagccccgtttacctg	CTACACGACGCTCTTCCGATCTctcagccccgtttacctg
SRSF2	Reverse	CAGACGTGTGCTCTTCCGATCT	ctgaggacgctatggatg	CAGACGTGTGCTCTTCCGATCTctgaggacgctatggatg
CALR	Forward	CTACACGACGCTCTTCCGATCT	GCAGCAGAGAAACAAATGAA	CTACACGACGCTCTTCCGATCTGCAGCAGAGAAACAAATGAA
CALR	Reverse	CAGACGTGTGCTCTTCCGATCT	GGACATCTTCCTCCTCATCT	CAGACGTGTGCTCTTCCGATCTGGACATCTTCCTCCTCATCT
POLQ exon14	Forward	CTACACGACGCTCTTCCGATCT	taaataaatgtgaaaccctttgt	CTACACGACGCTCTTCCGATCTtaaataaatgtgaaaccctttgt
POLQ exon14	Reverse	CAGACGTGTGCTCTTCCGATCT	caccagtcttgtgctattagatt	CAGACGTGTGCTCTTCCGATCTcaccagtcttgtgctattagatt
POLQ exon16	Forward	CTACACGACGCTCTTCCGATCT	tgtttgataaccttgaagaaaac	CTACACGACGCTCTTCCGATCTtgtttgataaccttgaagaaaac
POLQ exon16	Reverse	CAGACGTGTGCTCTTCCGATCT	gaagagcttagtccaacagaac	CAGACGTGTGCTCTTCCGATCTgaagagcttagtccaacagaac
POLQ exon18	Forward	CTACACGACGCTCTTCCGATCT	ctgcattattgatgctaagtaaa	CTACACGACGCTCTTCCGATCTctgcattattgatgctaagtaaa
POLQ exon18	Reverse	CAGACGTGTGCTCTTCCGATCT	ttttaaagaaattagtgccagtt	CAGACGTGTGCTCTTCCGATCTttttaaagaaattagtgccagtt
LIG4	Forward	CTACACGACGCTCTTCCGATCT	taaatcctttatgatcatccgta	CTACACGACGCTCTTCCGATCTtaaatcctttatgatcatccgta
LIG4	Reverse	CAGACGTGTGCTCTTCCGATCT	ccctcaaacttttaaactacaga	CAGACGTGTGCTCTTCCGATCTccctcaaacttttaaactacaga

Supplementary Table 5. Full description of NGS primers and sgRNA guides that were used throughout this manuscript. **a,** sgRNA sequences that were used to target *ASXL1* (Fig. 4a), *SRSF2* (Fig. 4b) and *CALR* (Supplementary Fig. 3) genes. Sequences in bold were used as synthetic sgRNAs targeting *ASXL1* and *SRSF2* genes throughout the manuscript. **b,** sgRNA sequences that were used to generatre knockout lines for *LIG4* and *POLQ* genes. **c,** NGS primers that were used throughout the manuscript.
