# Supplementary material for: Recurrent deletions in clonal hematopoiesis are driven by microhomology-mediated end joining
Source: Nat Commun. 2021 Apr 28;12:2455. doi: 10.1038/s41467-021-22803-y (PMC8080710; doi:10.1038/s41467-021-22803-y)
Supplement: Supplementary file 3 — Reporting Summary [file 41467_2021_22803_MOESM3_ESM.pdf]

nature research

Corresponding author(s): Dr. Liran Shlush
Last updated by author(s): Feb 24, 2021

Reporting Summary

Nature Research wishes to improve the reproducibility of the work that we publish. This form provides structure for consistency and transparency in reporting. For further information on Nature Research policies, see our Editorial Policies and the Editorial Policy Checklist.

Statistics

For all statistical analyses, confirm that the following items are present in the figure legend, table legend, main text, or Methods section.

n/a	Confirmed	
☐	☒	The exact sample size (*n*) for each experimental group/condition, given as a discrete number and unit of measurement
☐	☒	A statement on whether measurements were taken from distinct samples or whether the same sample was measured repeatedly
☐	☒	The statistical test(s) used AND whether they are one- or two-sided<br>*Only common tests should be described solely by name; describe more complex techniques in the Methods section.*
☐	☒	A description of all covariates tested
☐	☒	A description of any assumptions or corrections, such as tests of normality and adjustment for multiple comparisons
☐	☒	A full description of the statistical parameters including central tendency (e.g. means) or other basic estimates (e.g. regression coefficient) AND variation (e.g. standard deviation) or associated estimates of uncertainty (e.g. confidence intervals)
☐	☒	For null hypothesis testing, the test statistic (e.g. *F*, *t*, *r*) with confidence intervals, effect sizes, degrees of freedom and *P* value noted<br>*Give P values as exact values whenever suitable.*
☒	☐	For Bayesian analysis, information on the choice of priors and Markov chain Monte Carlo settings
☒	☐	For hierarchical and complex designs, identification of the appropriate level for tests and full reporting of outcomes
☐	☒	Estimates of effect sizes (e.g. Cohen's *d*, Pearson's *r*), indicating how they were calculated

Our web collection on statistics for biologists contains articles on many of the points above.

Software and code

Policy information about availability of computer code

Data collection	No software was used
Data analysis	Fastq files were aligned to hg19 human genome by using Minimap 2.1 (https://github.com/lh3/minimap2). Sorting and indexing of the SAM files were performed by using pysam 0.15.1 (https://github.com/pysam-developers/pysam). Assignment to new read groups were done by picard 2.8.3 (http://broadinstitute.github.io/picard). Local realignment was preformed using GATK3.7 (http://www.broadinstitute.org/gsa/wiki/index.php/The_Genome_Analysis_Toolkit). Mpileup files were generated by samtools 1.8 (https://github.com/samtools/samtools/) followed by indels and SNVs calling using VarScan 2.3.9 (https://github.com/dkoboldt/varscan).<br>In-house matlab code that was used for MMEJ detection is available at: https://github.com/ShlushLab/MMEJ_detection.<br>Metacell analysis code is available at: https://github.com/tanaylab/metacell/.<br>Other data analyses were performed with R version 3.5.2 (https://www.r-project.org/).

For manuscripts utilizing custom algorithms or software that are central to the research but not yet described in published literature, software must be made available to editors and reviewers. We strongly encourage code deposition in a community repository (e.g. GitHub). See the Nature Research guidelines for submitting code & software for further information.

Data

Policy information about availability of data

All manuscripts must include a data availability statement. This statement should provide the following information, where applicable:

- Accession codes, unique identifiers, or web links for publicly available datasets
- A list of figures that have associated raw data
- A description of any restrictions on data availability

Raw Illumina sequencing reads associated with CRISPR/Cas9 cell line experiments have been deposited in the NCBI Short Read Archive under bioproject ID

PRJNA707245 [https://www.ncbi.nlm.nih.gov/bioproject/707245]. All relevant data are also available from the corresponding author upon reasonable request. Publicly available datasets used in this study are available in the following web links: https://cancer.sanger.ac.uk/cosmic/download (COSMIC dataset), https://www.nejm.org/doi/full/10.1056/nejmoa1516192 (1540 adult-AML dataset), https://www.nejm.org/doi/full/10.1056/NEJMoa1716614 (2045 MPN dataset), http://www.vizome.org/aml/ (BeatAML dataset). Full data containing the deletion signatures from the publicly available datasets as well as CRISPR/Cas9 indel data are provided as a Source Data file.

Field-specific reporting

Please select the one below that is the best fit for your research. If you are not sure, read the appropriate sections before making your selection.

- [x] Life sciences
- [ ] Behavioural & social sciences
- [ ] Ecological, evolutionary & environmental sciences

For a reference copy of the document with all sections, see nature.com/documents/nr-reporting-summary-flat.pdf

Life sciences study design

All studies must disclose on these points even when the disclosure is negative.

Sample size	For deletion signature analyses from publicly available data, the largest relevant cohorts were chosen. As no statistical methods were performed, there was no need to predetermine sample size. In vitro experiments were performed in three biologically independent samples.
Data exclusions	No data were excluded from the analysis.
Replication	Three biological replicates were used in each experiments. The results were successfully replicated.
Randomization	The experiments were not randomized.
Blinding	The investigators were not blinded to allocation during experiments and outcome assessment.

Reporting for specific materials, systems and methods

We require information from authors about some types of materials, experimental systems and methods used in many studies. Here, indicate whether each material, system or method listed is relevant to your study. If you are not sure if a list item applies to your research, read the appropriate section before selecting a response.

Materials & experimental systems

n/a	Involved in the study	
☐	☒	Antibodies
☐	☒	Eukaryotic cell lines
☒	☐	Palaeontology and archaeology
☐	☒	Animals and other organisms
☐	☒	Human research participants
☒	☐	Clinical data
☒	☐	Dual use research of concern

Methods

n/a	Involved in the study	
☒	☐	ChIP-seq
☒	☐	Flow cytometry
☒	☐	MRI-based neuroimaging

Antibodies

Antibodies used

The following antibodies were used in this study:
anti-CD28 antibody (clone CD28.2, ThermoFisher scientific, 16-0289-81)
anti-CD3 antibody (clone OKT3, ThermoFisher scientific, 16-0037-81)
anti-CD45RA-FITC (BD Biosciences cat. no. 555488)
anti-CD38-PE-Cy7 (BD Biosciences cat. no. 335790)
anti-CD10-Alexa-700 (BD Biosciences cat. no. 624040)
anti-CD7-Pacific Blue (BD Biosciences cat. no. 642916)
anti-CD45-V500 (BD Biosciences cat. no. 560777)
anti-CD34-APC-Cy7 (custom made by BD, CD34 clone 581)
anti-CD34-PerCP-Efluor 710 (e-Bioscience 46-0344-42)
anti-CD33-PC5 (Beckman Coulter PNIM2647U)
anti-CD19-PE (BD Biosciences cat. no. 340364)
anti-CD3-FITC (BD Biosciences cat. no. 349201)
anti-CD56-Alexafluor 647 (BD Biosciences cat. no. 557711)
Streptavidin-QD605 (Invitrogen Q10101MP)
anti-CD8-APC-H7 (BD Biosciences cat. no. 560179)

	anti-light-chain lambda-V450 (BD Biosciences cat. no. 561379)<br>anti-light-chain kappa-V450 (BD Biosciences cat. no. 561327)<br>anti-CD57-APC (BD Biosciences cat. no. 555518)<br>anti-CD45-APC (BD Biosciences cat. no. 340943)
Validation	All antibodies were commercially validated for use in flow cytometry on human cells by the manufacturers Thermofisher, BD Biosciences, Invitrogen, Beckman Coulter and e-Bioscience. Full validation statements available on manufacturer's websites.

Eukaryotic cell lines

Policy information about cell lines

Cell line source(s)	K562, Marimo, MOLM-14, OCI-AML2 and OCI-AML3 cell lines were used in this study. All cell lines were obtained from ATCC
Authentication	whole exome sequencing was done to validate all cell lines
Mycoplasma contamination	All cell lines were tested negative for mycoplasma
Commonly misidentified lines (See ICLAC register)	No commonly misidentified lines were used in this study.

Animals and other organisms

Policy information about studies involving animals; ARRIVE guidelines recommended for reporting animal research

Laboratory animals	Eight to 12-week-old female NOD/SCID/IL-2Rgc-null (NSG) mice were used in this study. All mice were maintained under a 12 hours dark/light cycle, at an ambient temperature of around 22 degrees and humidity of 50%.
Wild animals	This study did not involve wild animals.
Field-collected samples	This study did not involve samples collected from the field.
Ethics oversight	Animal experiments were performed in accordance to the IACUC of the Weizmann Institute, its relevant guidelines and regulations (11790319-2).

Note that full information on the approval of the study protocol must also be provided in the manuscript.

Human research participants

Policy information about studies involving human research participants

Population characteristics	This study involved primary human samples derived from 13 individuals. Median age was 63 and 46% were female. Additional clinical characteristics are reported in Supplementary Tables 2, 3, 4.
Recruitment	All primary samples were pre-existing and obtained from the Leukemia Tissue Bank at Princess Margaret Cancer Centre. We do not know of any selection biases during the recruitments of these samples.
Ethics oversight	Recruitment was done according to procedures approved by the Research Ethics Board of the University Health Network (REB 01-0573-C), by the University health network ethics committee protocol # 15-9633, and Weizmann institute of science IRB protocol #337-1.

Note that full information on the approval of the study protocol must also be provided in the manuscript.
